# Applications of Metal–Organic Frameworks and Their Derivatives in Fuel Cells

**DOI:** 10.3390/molecules30050981

**Published:** 2025-02-20

**Authors:** Hongbiao Ling, Baoqiang Tian, Xiaoyan Hu, Weixu Wang, Jiaxing Zhang, Rui Liu, Zhen Lu, Yong Guo, Haidong Zhao

**Affiliations:** 1 School of Chemistry and Chemical Engineering, Shanxi Datong University, Datong 037009, China; linghongbiao2024@163.com (H.L.); 18602329176@163.com (X.H.); wang_wx10@163.com (W.W.); 13509735644@163.com (J.Z.); liurui198689@163.com (R.L.); 03110037@sxdtdx.edu.cn (Z.L.); 101000808@sxdtdx.edu.cn (Y.G.); 2 Shanxi Province Union Laboratory of Clean Energy Materials, Shanxi Datong University, Datong 037009, China; 3 Shanxi Center of Technology Innovation for Advanced Power Battery Material, Shanxi Normal University, Taiyuan 030031, China; xiaobao110@163.com

**Keywords:** metal–organic frameworks, derivatives, fuel cells, catalysts, supports, membranes

## Abstract

Metal–organic frameworks (MOFs) and their derivatives represent a novel class of porous crystalline materials characterized by exceptional porosity, high specific surface areas, and uniquely tunable physicochemical properties. These attributes render them highly promising for applications in the field of fuel cells. This review provides a comprehensive overview of the classification of MOFs and their current applications as catalysts, catalyst supports, and membranes in fuel cells. Additionally, the potential prospects and challenges associated with using MOFs and their derivatives in fuel cells are discussed, aiming to advance their development and offer valuable insights for researchers in this field.

## 1. Introduction

The lingering effects of the global energy crisis may herald the end of the fossil fuel era. However, modern society remains heavily dependent on fossil fuels. By the mid-21st century, global energy consumption is estimated to double, exacerbating the already critical energy challenges [1]. At the same time, the extensive use of fossil fuels has imposed unprecedented pressure on environmental protection. Developing efficient and sustainable green energy conversion technologies is crucial for addressing energy and environmental issues [2,3,4]. As a key representative of clean energy, fuel cells hold significant promise in the energy sector. They are poised to become a compelling alternative to fossil fuels [5]. Fuel cells are highly efficient and clean electrochemical devices that directly convert chemical energy into electrical energy. They are widely regarded for their high energy density, low emissions, and long operational lifespan. In recent years, growing global demand for clean energy has driven rapid advancements in fuel cell technology, enabling its application across various fields. Nevertheless, the technology faces critical limitations, including high costs and susceptibility to noble metal catalyst poisoning, fuel cell membrane (proton exchange membranes) instability, and challenges associated with oxygen reduction reaction (ORR) kinetics. Therefore, developing efficient, stable, and cost-effective fuel cell catalysts and membranes, coupled with efforts to lower reaction energy barriers and enhance reaction rates, is essential for improving energy conversion efficiency [6].

**Figure 1:**
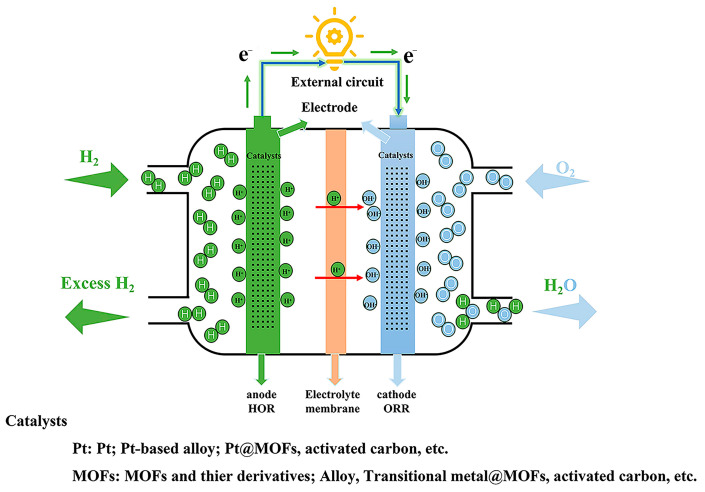
A schematic diagram of a fuel cell, with an anode on the left where hydrogen oxidation reaction occurs and a cathode on the right where oxygen reduction reactions take place.

Fuel cells primarily consist of three components: electrodes, an electrolyte membrane, and an external circuit (Figure 1). Electrodes are divided into anodes and cathodes. The anode, also called the fuel electrode, serves as the site where fuel is introduced and catalyzed during electrochemical reactions. At the same time, the cathode provides the environment for fuel oxidation [7,8,9]. Metal–organic frameworks (MOFs), a novel class of porous crystalline materials, are constructed by linking metal ions or clusters with organic ligands through coordination bonds. These materials exhibit exceptional properties, such as high specific surface areas, large pore volumes, robust physicochemical stability, and excellent conductivity. As a result, MOFs have found extensive applications in areas such as gas production and storage, electrode material preparation, catalysis, and energy storage and conversion technologies [10,11].

Moreover, recent studies have demonstrated that MOF derivatives can function directly as catalysts or as catalyst supports. By providing a larger specific surface area, they enhance the utilization of catalytic active sites and reduce the overall cost of catalyst usage [12,13]. As shown in Figure 1, MOFs and their derivatives can be attached to the electrode (cathode or anode) as a fuel cell catalyst or catalyst carrier to promote electrochemical reactions on the electrode’s surface. For example, Li et al. [1] prepared trimetallic MOFs based on Fe/Ni (Fe/Ni/Co(Mn)-MIL-53). Fe/Ni/Co(Mn)-MIL-53 exhibits volcano-type oxygen evolution reaction (OER) activity as a function of its composition. The optimized Fe/Ni_2.4_/Co_0.4_-MIL-53 can achieve a current density of 20 mA·cm^−2^ at a low overpotential of 236 mV, with a small Tafel slope of 52.2 mV·dec^−1^. This demonstrates its significant activity as an OER catalyst, providing new insights for applying MOFs as catalysts in fuel cells. In addition, the B-doped graphene quantum dots and bimetallic MOF composite catalysts developed by Yan et al. [14] have a maximum power density of 703.55 mW·m^−2^ in a microbial fuel cell (MFC), which is 1.53 times that of the Pt/C cathode. The catalyst cathode maintained good stability for 800 h, while the stability of the Pt/C cathode dropped to 61% of the initial voltage. The experimental results showed that the catalyst has excellent oxygen reduction reaction (ORR) activity and excellent electrochemical stability. These findings highlight MOFs’ adaptability and promising potential in designing fuel cell catalysts, paving the way for their broader applications in this field. However, when used as catalysts, most original MOFs still face problems, such as poor electrical conductivity, insufficient chemical and thermal stability, and low catalytic activity. Current methods for solving these problems mainly include combining MOFs with other nanostructures (such as graphene and nanotubes), growing MOFs directly on conductive electrode substrates, pyrolyzing MOF derivatives to obtain MOFs, and introducing MNPs into MOFs as guest active sites to solve the above problems, thereby enabling MOFs to be better applied in the field of fuel cells [15,16,17,18].

As research progresses, MOFs are set to assume an increasingly significant role in the field of fuel cell technology. This paper provides a comprehensive review of recent advancements in the research and development of MOFs and their derivatives in the context of fuel cell catalysts, catalyst supports, and fuel cell membranes. The review offers valuable insights and is a valuable reference point for researchers operating within related domains.

## 2. Classification of MOFs

MOFs are a novel class of porous crystalline and inorganic–organic hybrid materials [19]. MOFs encompass a wide variety of structures, and their classification methods are equally diverse. In this review, the classification is primarily based on their constituent units [20].

### 2.1. Zeolitic Imidazolate Frameworks (ZIFs)

Zeolitic imidazolate frameworks (ZIFs) are a specialized subclass of MOFs. They consist of tetrahedrally coordinated transition metal ions (e.g., zinc (divalent) or cobalt (divalent)) and nitrogen atom linkers on imidazoles to form porous crystalline materials with a zeolite topology, with the tetrahedral and bridging Si(Al) units consisting of transition Si(Al) units and imidazole ligands, respectively. ZIF exhibits a molecular sieve network structure of sodalite, which affords it a substantial specific surface area and porosity. Consequently, it has the capacity to enhance the mechanical properties, proton conductivity, and ion conductivity of membranes significantly [21]. ZIFs have a wide variety of structures, including ZIF-7 [22], ZIF-8 [23], ZIF-11 [23], and ZIF-67 [24]. Among these, ZIF-8 and ZIF-67 are representative materials that have been extensively studied and developed in fuel cell research. For example, Rong et al. [21] designed and synthesized a series of transition metal nanoparticle composites supported on ZIF-67 (TMNPs/ZIF-67), including platinum (Pt), palladium (Pd), silver (Ag), gold (Au), rhodium (Rh), and rhenium (Re). These transition metal composites were primarily utilized in selective hydrogen and oxygen production reactions. Among them, the optimized Pt/ZIF-67 nanocatalyst showed excellent performance in ammonia borane hydrolysis for hydrogen production and hydrogen peroxide decomposition for oxygen production. The key is that the “on-off” control of ammonia borane hydrolysis for hydrogen production is achieved through the Zn^2+^/EDTA-2Na system, which provides a solid technical foundation for the development of fuel cells. Patil et al. [25] utilized microwave-assisted synthesis to prepare copolymer membranes from polyvinyl alcohol (PVA) and acrylic acid (AA), with varying contents of ZIF-8 nanoparticles (1, 2.5, 5, and 10 wt.%) as fillers. The experimental findings demonstrated that as the content of ZIF-8 in the membrane augmented, so did its ion exchange capacity, consequently enhancing proton conductivity. The proton conductivity of these membranes could reach up to 3.33 × 10^−2^ S·cm^−1^, which was a significant finding. In addition, the membranes demonstrated good permeability and thermal stability, making them suitable for use in the research and application of fuel cells.

### 2.2. University of Oslo (UiO)

The UiO series refers to a class of MOFs first reported by researchers at the University of Oslo, from which the name “UiO” is derived. The UiO frameworks are constructed from Zr^4^^+^ ions coordinated with dicarboxylate organic linkers via coordination bonds, resulting in materials with high crystallinity and porosity. These materials exhibit highly tunable chemical properties, which can be modulated through the choice of various organic linkers [26]. The different structures and properties of the organic linkers lead to significant variations in the pore architecture and chemical characteristics of the UiO series. Currently, the UiO series primarily includes three types: UiO-66, UiO-67, and UiO-68 [27]. These three materials have been extensively studied and applied due to their uniform pore sizes, high porosity, excellent thermal stability, and large specific surface areas [28]. While these materials share similar structures, they differ in their linkers: terephthalate for UiO-66, biphenyl dicarboxylate for UiO-67, and terphenyl dicarboxylate for UiO-68 [29]. Considering that increasing the number of benzene rings in the linker significantly raises the cost of MOF synthesis, UiO-66 offers better economic efficiency. Momeni and colleagues [30] employed a highly efficient electrocatalyst (Ni(OH)_2_-UiO-66/CPE) modified with UiO-66 nanoparticles for direct borohydride fuel cells (DBFCs). The findings demonstrated that the electrode modified with Ni(OH)_2_-UiO-66/CPE exhibited a catalytic rate and diffusion coefficient of 1.53 × 10^3^ cm^3^·mol^−1^·s^−1^ and 1.51 × 10^−7^ cm^2^·s^−1^, respectively, for the oxidation of sodium borohydride in an alkaline medium. Furthermore, the area current density was found to be 91% and 77.9% of the initial value after 14 and 90 days, respectively, indicating that the introduction of UiO-66 enhanced the catalytic activity of the DBFC catalyst and its stability. This research has significantly advanced the development of direct borane sodium fuel cells. Additionally, Liu et al. [31] modified UiO-66 by introducing functional groups such as H, NH_2_, Br, and I to investigate the relationship between platinum catalyst activity and linker types. Their findings revealed that when Pt atoms were anchored onto the modified UiO-66-Br framework, the catalyst exhibited the highest activity and excellent thermal stability, with no metal aggregation observed even at 300 °C. The successful introduction of sulfonic acid into the zirconium-based MOF UiO-66 was realized by a “click” reaction. The resulting MOF, UiO-66-SO_3_H, exhibited a significant increase in proton conductivity at 80 °C and 98% relative humidity, reaching 8.8 × 10^−3^ S cm^−1^. This is a significant improvement over the proton conductivity of pure UiO-66 and UiO-66-NH_2_ under the same conditions (6.3 × 10^−6^ and 3.5 × 10^−6^ S·cm^−1^, respectively). The click-modified UiO-66-SO_3_H also exhibited excellent long-term stability and reusability, making it a promising candidate for proton exchange membranes in fuel cell applications [32].

### 2.3. Isoreticular Metal–Organic Frameworks (IRMOFs)

Isoreticular metal–organic frameworks (IRMOFs) represent a class of MOFs with similar framework topologies. These microporous crystalline materials are constructed through the self-assembly of isolated secondary building units and [Zn_4_O]^6^^+^ inorganic clusters, with a series of aromatic carboxylate ligands in an octahedral arrangement. A defining feature of IRMOFs is their ability to maintain identical topological structures, such as pore size, specific surface area, stability, and gas adsorption properties, while allowing the framework to be tuned by varying the metal ions (e.g., Zn^2^^+^, Cu^2^^+^, Mg^2^^+^) or the size and chemical structure of the organic linkers (e.g., benzene-dicarboxylate or terephthalate) [33,34]. Prominent examples of IRMOFs include IRMOF-1, IRMOF-3, IRMOF-5, IRMOF-8, and IRMOF-10 [34]. The design concept of the IRMOF series endows these materials with exceptional performance in terms of porosity and specific surface area. By adjusting the structure of organic linkers, the pore architecture of IRMOFs can be precisely controlled, often achieving extremely high specific surface areas (typically exceeding 2000 m^2^·g^−1^). This makes them highly versatile for applications in gas storage [35], catalysis [36], and sensing [37]. For example, IRMOF-1 (also known as MOF-5) has demonstrated significant potential for hydrogen storage, which has, to some extent, advanced fuel cell development by addressing the limitations of hydrogen storage. Additionally, derivatives of MOF-5 have shown promising applications in catalysis. Sun and his colleagues [38] utilized MOF-5-derived carbon-coated ZnO as catalyst support to load Pd nanoparticles with the objective of enhancing the stability and anti-toxicity of the catalyst. The research findings demonstrated that the catalyst Pd/ZnO/MDC, obtained at an annealing temperature of 800 °C, exhibited a mass activity of 931.83 mA·mg^−1^ for methanol oxidation reactions (MORs) and 1838.30 mA·mg^−1^ for ethanol oxidation reactions (EORs), which is 2.4 and 1.8 times that of Pd/C (383.72 mA·mg^−1^ and 977.22 mA·mg^−1^), achieving the efficient catalysis of MOR and EOR. This research not only provides a feasible idea for the design and production of fuel cell catalysts but also lays a better theoretical foundation for the development of fuel cells.

### 2.4. Material Institut Lavoisier (MIL)

The Material Institut Lavoisier (MIL) series represents a well-known family of MOFs developed by the Institut Lavoisier de Versailles in France. These porous crystalline materials are composed of various trivalent metal cations and carboxylate ligands characterized by their large voids and permanent porosity [39]. The MIL series includes a variety of specific compounds, typically designated as MIL-XXX, where “XXX” refers to the specific chemical structure number. Representative examples include MIL-53, MIL-68, MIL-88, and MIL-100 [40]. The MIL series of MOFs exhibits high stability, permanent porosity, and exceptionally large specific surface areas. With the advancement of fuel cell technology, MIL-series MOFs have garnered significant attention from researchers and have been explored as catalysts for fuel cells. For instance, Wang and his colleagues [41] utilized MIL-53 as a cathode catalyst for MFC, with the results indicating that the maximum current density of MIL-53 (0.05 mA) was 10 times that of the blank electrode (0.005 mA). This suggested that the introduction of MIL-53 significantly enhanced the ORR activity of the MFC. Concurrently, the maximum power density of the MFC was determined to be 397 ± 6.3 mW·m^−2^, which was 73.5 times that of the bare electrode (5.4 mW·m^−2^). In addition, Akin and colleagues [42] synthesized a Pt@MIL-125 (Ti) catalyst using a solvothermal method by combining MIL-125 (Ti) with Pt nanoparticles (NPs) for the MOR in direct methanol fuel cells (DMFCs). The results revealed that Pt@Ti-MOF NPs exhibited electrocatalytic activity for methanol oxidation that was 9.45 times higher than that of Pt NPs, while also significantly improving the stability and durability of Pt NPs.

In addition to the previously mentioned ZIF, UiO, IRMOF, and MIL series, there exist numerous other types of MOF materials, including but not limited to the Coordination Pillared-Layer (CPL) series, Porous Coordination Network (PCN) series, Hong Kong University of Science and Technology (HKUST) series, and Nanyang Technological University (NTU) series. These MOFs have been extensively studied and applied due to their unique structures and physicochemical properties. It is anticipated that with advancements in science and technology, more MOF materials will emerge, offering new possibilities for the development of fuel cell technologies.

## 3. MOFs and Their Derivatives as Fuel Cell Catalysts

**Figure 2:**
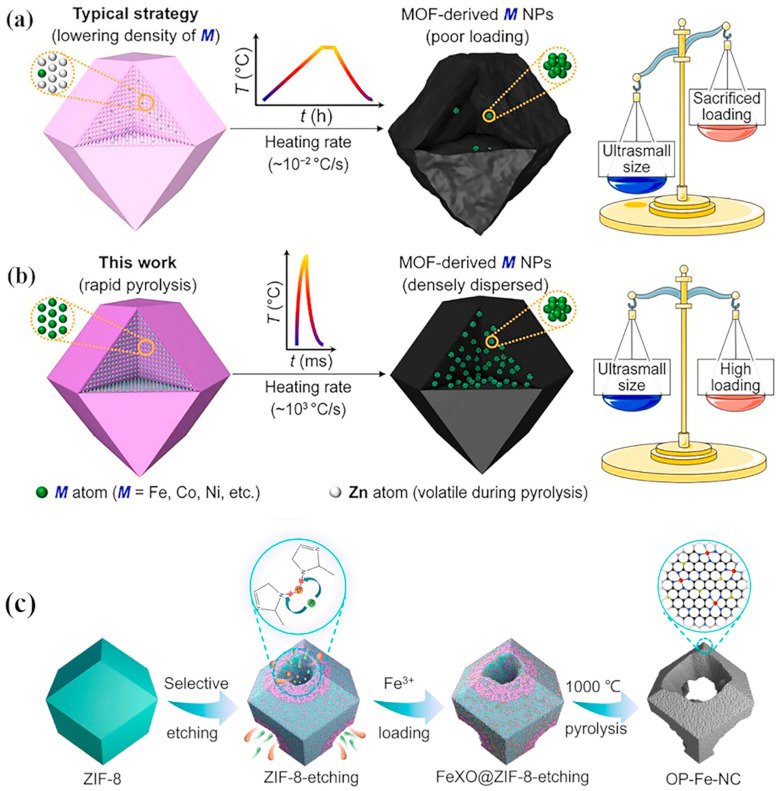
Comparison of pyrolysis methods for MOFs. (**a**) The most common pyrolysis method; (**b**) millisecond pyrolysis method [52], copyright 2022 Elsevier; (**c**) pyrolysis method after etching [53]. Copyright 2023 ACS Publications.

MOF materials exhibit high porosity, crystallinity, and large specific surface areas, making them promising candidates as catalysts for electrocatalytic reactions. Recently, they have been engineered to include secondary building blocks, large pore sizes, open metal sites, and functional precursors to enhance electronic conductivity. These materials possess considerable potential in fuel cell applications and can be enhanced through various ways to improve their electronic transport properties [43]. However, most MOFs exhibit high charge transfer barriers and limited free charge carriers [44,45,46], which results in poor conductivity, thus hindering their direct application in electrocatalysis [47]. Additionally, MOFs may face structural stability issues during electrocatalytic processes, where catalysts are susceptible to corrosion or structural changes, leading to a decrease in catalytic activity and impairing electrochemical reactions [48,49]. To address these challenges, researchers have proposed several strategies to enhance the electrochemical performance of MOFs. Firstly, the incorporation of conductive polymers or carbon materials can significantly improve the conductivity of MOFs [50,51]. Secondly, tuning the pore structure and morphology of MOFs can increase the number of active sites and reduce ion transport resistance [6]. Furthermore, combining MOFs with nanoparticles or metal oxides can further optimize their electrochemical properties [19]. Additionally, methods such as millisecond pyrolysis [52], pyrolysis after etching [53], or doping modifications [31,54] can be employed to convert MOFs into materials suitable for direct or indirect electrochemical applications (see Figure 2).

**Figure 3:**
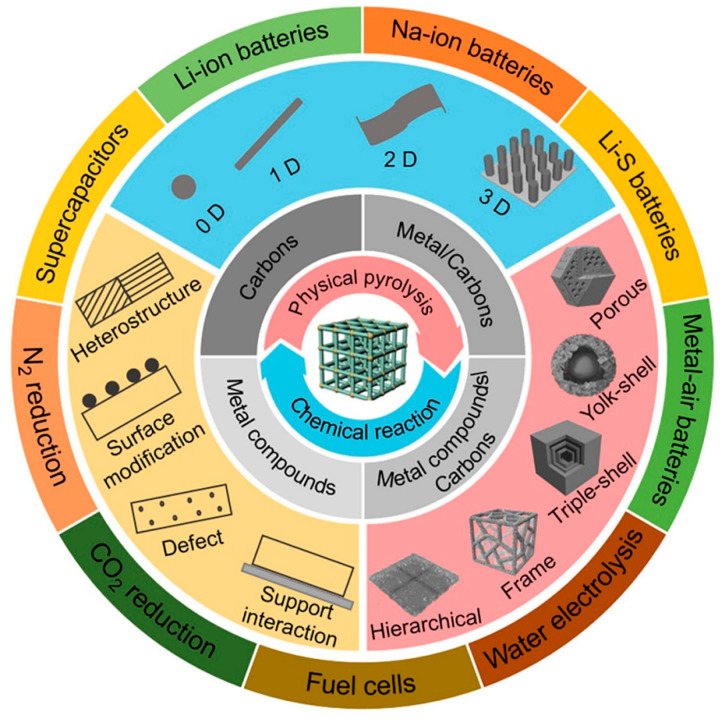
Synthetic strategies of MOF-derived functional materials with different compositions and structures, performance improvement approaches, and their applications in various EESC systems [58]. Copyright 2021, Nano Letters.

As a new class of porous materials, MOFs and their derivatives have demonstrated enormous potential for applications in electrochemical energy storage and conversion. Through rational synthesis strategies and performance optimization, MOFs have exhibited outstanding electrochemical properties in supercapacitors [55], lithium-ion batteries [56], zinc-ion batteries [57], fuel cells [11], and other electrochemical energy storage and conversion devices [58] (see Figure 3).

In fuel cell reactions, the main ORR and hydrogen oxidation reaction (HOR) are the core technologies of fuel cells and are necessary for the conversion of chemical energy into electrical energy. The ORR is a cathodic reaction, and the HOR is an anodic reaction. The two reactions occur in the same environment to form a specific cell [54,59,60]. In these reactions, MOFs can provide catalytic active sites, and their structural and functional group tunability also makes it possible to enhance specific reaction activities [59]. MOF derivatives are attracting attention due to the excellent thermal and chemical stability of metal nanoparticles or metal oxide nanoparticles. Furthermore, MOF derivatives retain the typical topological structure, morphology, ultra-high surface area, and rich porosity of the sacrificial precursor [61,62,63]. This not only improves the electronic conductivity of the material but also allows metal nanoparticles to be embedded in porous carbon doped with heteroatoms, thereby protecting the metal nanoparticles from aggregation and corrosion, resulting in ideal catalytic/co-catalytic activity and stability [64,65,66].

In summary, although MOFs still face challenges in practical applications, such as low conductivity and difficulties in electron transfer, their excellent reprocessing ability continues to make them widely used in electrochemical energy storage and conversion. They remain a highly promising material for future research in the field of electrochemistry.

### 3.1. MOFs and Their Derivatives as Direct Anode Catalysts for Fuel Cells

MOFs can be directly used as anode catalysts to enhance anode reaction activity. For example, materials such as MIL-53, MIL-88, MIL-100, ZIF-67, and NiFe-MOF have been explored for this purpose. Some MOFs’ derivatives can also be used directly as anode catalysts because they can provide unique nanomorphologies and more reaction sites to enhance catalytic reaction activity while significantly overcoming the chemical instability and poor electronic conductivity of MOFs. To further enhance anode reaction activities, a second or even a third metal ion can be deliberately introduced into MOFs to further change the coordination environment of the reaction active sites and the electronic transport environment [59].

**Figure 4:**
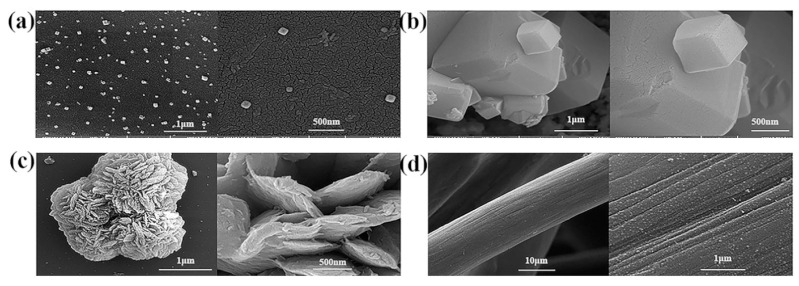
FE-SEM images of Co-MOF (**a**), Cu-MOF (**b**), CoCu-MOF (**c**), and T-CF (**d**) [67]. Copyright 2023, Elsevier.

**Figure 5:**
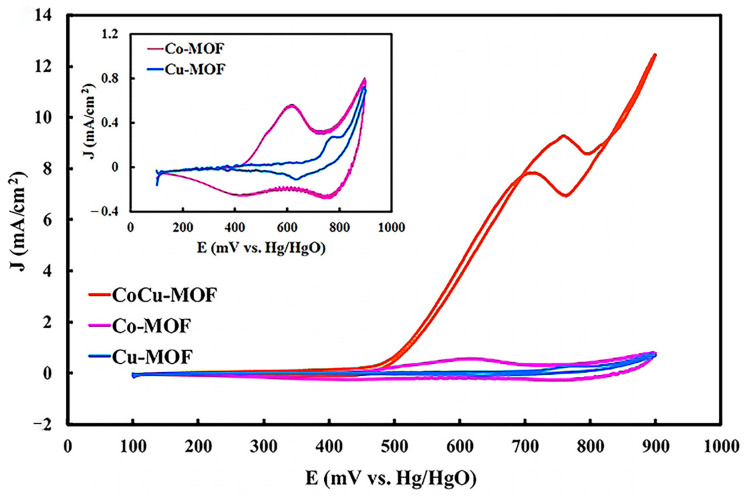
EOR CV curves in 0.5 M NaOH + 0.5 M C_2_H_5_OH at a scan rate of 20 mV s^−1^ [67]. Copyright 2023, Elsevier.

**Figure 6:**
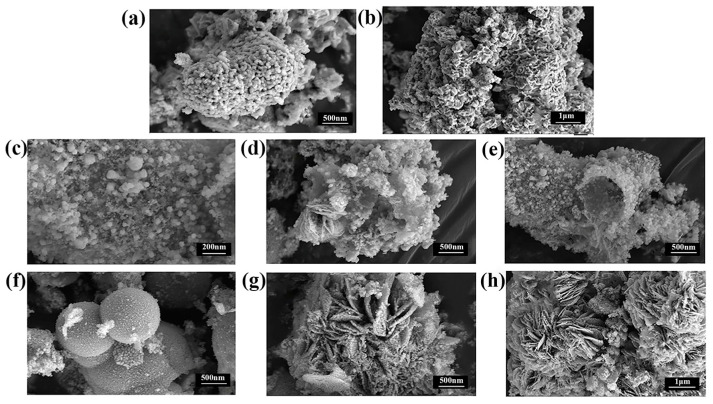
FE-SEM images (**a**,**b**) for MOFP1, (**c**–**e**) for MOFP2, and (**f**–**h**) for MOFP3 at different magnification scales [71]. Copyright 2024, Royal Society of Chemistry.

**Figure 7:**
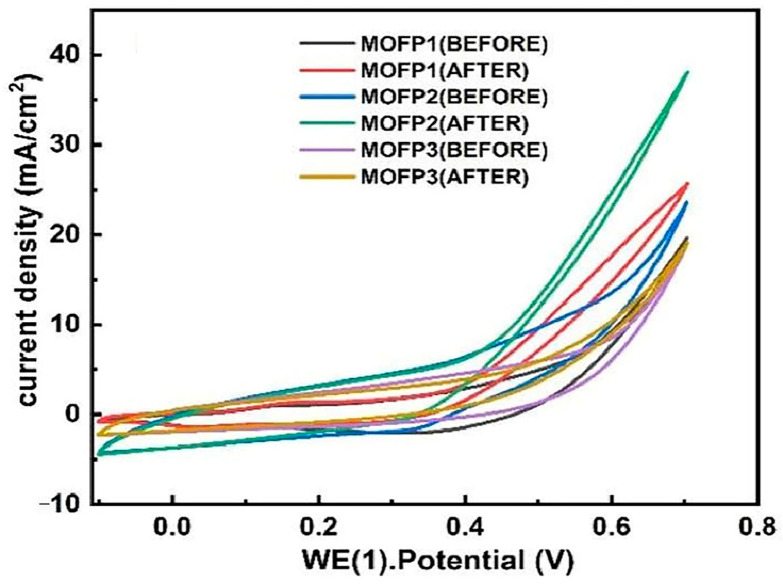
Cyclic voltammograms, CV, of MOFP composites without methanol, at 50 mV·s^−1^ CV [71]. Copyright 2024, Croya Society of Chemistry.

For instance, Mohammadi et al. [67] studied the performance of a flower-like CoCu-MOF loaded on carbon felt as a binder-free anode electrode in direct ethanol fuel cells (DEFC). Figure 4 presents the FE-SEM images of Co-MOF, Cu-MOF, CoCu-MOF, T-CF, and CoCu-MOF/CF. In Figure 4a,b, it is observed that Co-MOF exhibits a homogeneous cubic structure, while Cu-MOF displays a polyhedral surface with a smooth texture. Figure 4c illustrates that CoCu-MOF has a distinctive flower-like structure composed of interconnectedly sized petals. As shown in Figure 4d, the exposed T-CF features numerous neat carbon fibers devoid of organic impurities. Following the in situ growth of CoCu-MOF on the T-CF surface, a nanoflower-like structure with many edges and thinner petals is formed. This unique petal-like nanoflake structure enhances the specific surface area and provides more active sites, ultimately improving the electrochemical performance (see Figure 4). The results showed that CoCu-MOF exhibited a much higher current density for EOR than Co-MOF and Cu-MOF, demonstrating excellent electrocatalytic performance (see Figure 5). Sayed et al. [68] successfully synthesized Ni-MOF on nickel foam (NF) and improved its conductivity by adding reduced graphene oxide (rGO). Their findings demonstrated that Ni-MOF@NF and Ni-MOF-rGO@NF exhibited superior catalytic activity for EOR compared to pure nickel foam. Additionally, MOF derivatives can be converted into target catalysts through pyrolytic carbonization. Pyrolytic carbonization involves heating MOFs to high temperatures in an inert atmosphere (such as N_2_ or Ar) to remove organic components, leaving behind metallic elements, metal oxides, and carbon materials. The products obtained through pyrolysis effectively enhanced the stability of the catalysts [69,70]. Kamal et al. [71] first prepared Cu-MOF, Cu/Ni-MOF, and Cu/Ni/Co-MOF precursors using a simple hydrothermal method, followed by pyrolytic carbonization at 600 °C in a nitrogen atmosphere for two hours. The Cu-MOF complex (MOFP1), Cu/Ni-MOF complex (MOFP2), and Cu/Ni/Co-MOF complex (MOFP3) were obtained. Figure 6 shows the FE-SEM images of MOFP1, MOFP2, and MOFP3. MOFP1 is a nanoflower structure (Figure 6a,b), MOFP2 is a hollow nanoflower structure with an open bilayer structure that may enhance electrochemical properties (Figure 6c–e), and MOFP3 is a nanoflower structure with sharp, thinly coated aggregates and overall spherical clusters (Figure 6f–h). The electrochemical assay results showed that the Cu/Ni-MOF complex (MOFP2) exhibited the best performance in MOR, as shown in Figure 7, with a current density of 38.77 mA·cm^−2^ at a scan rate of 60 mV·s^−1^, demonstrating excellent electrochemical performance, which was attributed to its unique porous open flower structure and the synergistic effect between the metal components.

Both direct MOF catalysts and MOF-derived catalysts exhibit significant potential for use as anode catalysts in fuel cells. By optimizing and modifying the structure and composition of these catalysts, their catalytic activity can be greatly enhanced, thereby improving the overall performance of fuel cells.

### 3.2. MOFs and Their Derivatives as Direct Cathode Catalysts for Fuel Cells

**Figure 8:**
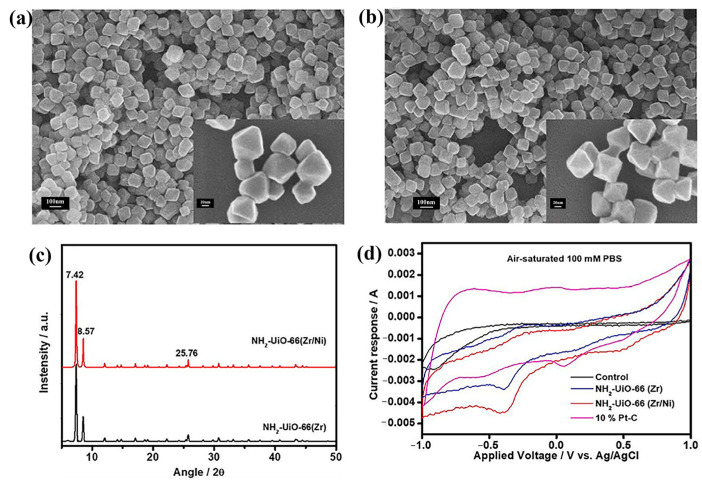
Materials properties: (**a**) FESEM micrograph of NH_2_-UiO-66 (Zr); in the inset, a magnified image at 500 kX at the 20 nm scale. (**b**) FESEM micrograph of NH_2_-UiO-66 (Zr/Ni); in the inset, a magnified image at 500 kX at the 20 nm scale. (**c**) PXRD analysis. (**d**) CV scan in air-saturated electrolytes with identified current peaks [74]. Copyright 2022, Elsevier.

MOFs and their derivatives, due to their unique structure and excellent performance, exhibit significant application potential in electrocatalysis, particularly in the ORR. Similarly, some MOFs can be directly used as cathode catalysts. For instance, Li et al. [72] introduced a series of conductive MOFs based on 2,3,6,7,10,11-hexahydroxytriphenylen (HHTP) synthesized with different metal centers, such as Cu, Zn, Co, Ni, and Mn. These MOF materials were evaluated as cathode catalysts for air-cathode MFC to enhance ORR performance. Among them, Cu-HHTP, with a three-dimensional coral-like structure, exhibited the best ORR catalytic activity. It achieved a maximum power output of 206.67 mW·m^−2^ and a stable voltage output of 275 mV in the air-cathode MFC, which is 2.25 times higher than that of the blank carbon cloth cathode. Chen et al. [73] conducted a density functional theory (DFT) study on the ORR activity of a series of M_3_(HHTT)_2_ MOFs (where M represents various 3d, 4d, and 5d transition metals). The results indicated that Fe_3_(HHTT)_2_, Co_3_(HHTT)_2_, Rh_3_(HHTT)_2_, and Lr_3_(HHTT)_2_ exhibit high ORR catalytic activity. These MOFs theoretically possessed a minimum overpotential of 0.21 V, outperforming Pt (111). Additionally, Co_3_(HHTT)_2_ demonstrated excellent poison resistance against impurity gases and fuel molecules. Although not yet implemented in practical applications, the success of theoretical research has laid a solid foundation for practical operations and provided additional theoretical support for the use of MOF materials in fuel cells. Noori et al. [74] also reported a novel bimetallic MOF catalyst, NH_2_-Uio-66(Zr/Ni). A comparison of NH_2_-Uio-66(Zr) and NH_2_-Uio-66(Zr/Ni) revealed that the introduction of the Ni element did not result in significant variations in their structural and morphological characteristics. As illustrated in Figure 8, both NH_2_-Uio-66(Zr/Ni) and NH_2_-Uio-66(Zr) manifested an octahedral structure (tridiagonal crystalline row state) with a uniform particle size distribution. In Figure 8c, a complete set of sharp diffraction peaks can be seen in the Bragg angle window of 5 to 50°. The dominant diffraction peaks at 7.4 and 8.5° correspond to d-spacings of 11.9 Å and 10.3 Å, respectively. The diffraction peaks of these two MFOs were consistent, indicating that they have the same crystal structure. However, their catalytic properties exhibited significant disparities. The results demonstrated that the incorporation of Ni into NH_2_-Uio-66(Zr) enhanced the molecular arrangement relative to the high electron crowding in the metal node clusters and improved the surface porosity, thereby facilitating the bimetallic NH_2_-. The bimetallic NH_2_-Uio-66(Zr/Ni) demonstrated superior performance in terms of ORR activity and MFC generation performance when compared to mono-metallic NH_2_-Uio-66(Zr) and commercial 10% Pt catalysts (see Table 1). As is clearly visible in Figure 8d, the CV current response obtained from NH_2_-UiO-66(Zr/Ni) catalyzed cathodes in both the N_2_- and air-saturated electrolytic medium at 10 mV·s^−1^, and the scan rate significantly surpassed the control cathode (no catalyst) and NH_2_-UiO-66(Zr)-catalyzed cathode; it was even better than that of the 10% Pt-C cathode. These bimetallic MOFs exhibited a high specific surface area, low charge resistance, and high diffusion coefficient, which enhanced ORR kinetics and MFC power density.

However, only a few MOFs can be directly used as cathode catalysts for fuel cells. To overcome this limitation, numerous researchers have focused on MOF-derived materials to develop various targeted catalysts. Considering the cost of Pt-based catalysts and the economic viability of fuel cell commercialization, some researchers have developed many non-pt group catalysts [75,76]. Examples include Zn/Co-N-C non-precious metal single-atom catalysts [77]; NiCo-supported metal–organic framework CPO-27 (NiCo-CPO-27); and graphite-like carbon nitride (g-C_3_N_4_) [78], Fe-N-C catalysts [79], 3D ordered macroporous nitrogen-doped carbon-encapsulated iron–nitrogen alloys (Fe/Fe-NA@NC), and others [80]. Among non-platinum group catalysts, metal–carbon–nitrogen (M-C-N) materials are the most common. These catalysts exhibited excellent ORR activity, high half-wave potential, peak power density, and outstanding stability. Their performance can rival or even surpass that of commercial Pt/C catalysts. The exceptional electrocatalytic performance of these MOF-derived materials is attributed to the direct influence of the active species in the MOF-derived carbon material (MDCNM) matrix during the catalysis process. Depending on the active species present, these catalysts can be classified into heteroatom-doped carbon-based catalysts, single-metal carbon-based catalysts, bimetallic/multimetallic carbon-based catalysts, and metal-composite carbon-based catalysts. These include non-metallic active centers (pyridinic N and graphitic N), metallic active centers (M-Nx), and other metallic active species (alloy nanoparticles and metal compounds) [81].

### 3.3. Platinum-Group Metal@MOF Composite Catalysts for Fuel Cells

**Figure 9:**
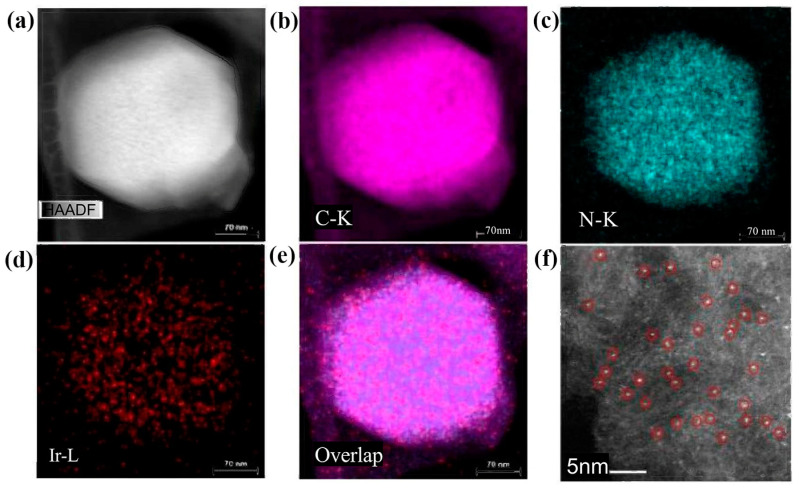
(**a**–**e**) STEM images and the corresponding elemental mappings for Ir-SAC; (**f**) high-resolution HAADF-STEM image of Ir-SAC, with the distinct bright dots (circled in red) indicating that Ir is atomically dispersed on the nitrogen-doped carbon matrix [84]. Copyright 2019, Wiley-VCH.

**Figure 10:**
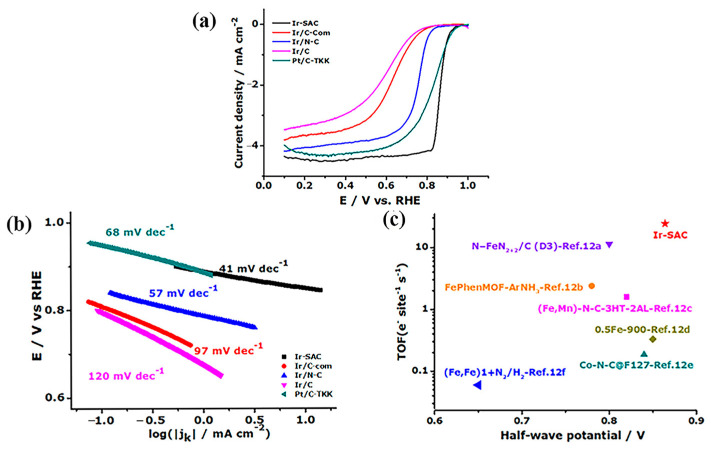
(**a**) ORR polarization curves with a scanning rate of 5 mV s^−1^ at a rotating speed of 900 rpm for the synthesized catalysts; (**b**) LSV curves of Ir-SAC with various rotation rates; (**c**) TOF and E_1/2_ values of Ir-SAC and other recently reported SACs, where the TOF values were estimated at the potential of 0.85 V for Ir-SAC and 0.8 V for the other catalysts [84]. Copyright 2019, Wiley-VCH.

Another group of researchers continues to focus on studying and developing Pt-based fuel cell catalysts. For example, Huang et al. [82] reported the development of a highly efficient Pt-nanocarbon integrated electrocatalyst, PtCo@CoNC/NTG, optimized using a multiscale design strategy for the ORR in fuel cells. The research results showed that the catalyst has a current density of 1.52 A·cm^−2^ and a maximum power density of 980 mW·cm^–2^ at 0.6 V vs. RHE, which is 1.25 times higher than that of commercial Pt/C (1.21 A·cm^−2^, 780 mW·cm^–2^) and has excellent stability. During the introduction of transition metal alloying, strain and coordination effects are induced, enhancing the catalytic activity of platinum [47]. Furthermore, this process not only reduces the required amount of Pt but also modulates the Pt-Pt bond distance [83], thereby improving the ORR performance of the catalyst. In addition to Pt-based catalysts, Ru-, Pd-, and Ir-based catalysts have also emerged as promising candidates for fuel cell cathode applications. For instance, Xiao et al. [84] employed ZIF-8 as an active site carrier and acetylacetonate iridium as the guest to synthesize a highly efficient single-atom iridium catalyst (Ir-SAC). As shown in Figure 9a, the well-preserved dodecahedral nanostructures and, more importantly, the absence of any observable Ir nanoparticles/nanoclusters were revealed via high-angle annular dark field scanning transmission electron microscopy (HAADF-STEM). However, the energy dispersive spectroscopy elemental distributions (Figure 9b–e) and inductively coupled plasma (ICP) results confirmed the presence of Ir in the resulting Ir-SACs, suggesting the presence of atomic-level Ir embedded in the carbon matrix. On this basis, the aberration-corrected HAADF-STEM was further utilized to identify the Ir distribution directly. According to Figure 9f, many speckled bright spots are displayed, which strongly confirms the atomic segregation of Ir in the obtained Ir-SAC. As shown in Figure 10b,c, Ir-SAC exhibited an excellent transition frequency (TOF) at 0.85 V vs. RHE in the ORR reaction of PEMFC, ahead of previously reported SAC and commercial Pt/C catalysts. The high catalytic activity is attributed to the moderate adsorption of the reaction intermediate on the coordination sites of mononuclear iridium ions with four nitrogen radicals. In addition, it is also mentioned in the report that Ir-SAC has an onset potential (E_onset_) of 0.97 V and a half-wave potential (E_1/2_) of 0.864 V in a real hydrogen–oxygen fuel cell (as shown in Figure 10a), which is superior to most non-Pt OOR catalysts in terms of open-circuit voltage and power density, suggesting that it has a promising future in fuel cell research. In the same year, Xiao et al. [85] synthesized a novel ruthenium-based single-atom site catalyst (Ru-SSC) by encapsulating a ruthenium precursor in the micropores of ZIF-8 using the MOF confinement strategy. The results showed that the in situ generated OH ligands adjusted the d orbital electronic structure of ruthenium and optimized the adsorption–desorption behavior of oxygen-containing intermediates, resulting in a Ru-SSC with a TOF of 4.99 es^−1^sites^−1^ and superior stability (only a negative shift of 17 mV after 20,000 cycles), which far exceeds the TOF (0.816 es^−1^sites^−1^) and stability (a negative shift of 31 mV after 20,000 cycles) of the most advanced Fe-SSC.

## 4. MOFs and Their Derivatives as Catalyst Supports for Fuel Cells

In addition to developing new catalysts, enhancing the interaction between platinum and supports is also vital for boosting catalytic efficiency and stability [86]. Reported catalyst supports for fuel cells primarily include materials such as activated carbon [87], zeolites [88], graphene [89], TiO_2_ [90], and MOFs, along with their derivatives. Compared to these support materials, MOFs and their derivatives have garnered significant attention from researchers due to their high specific surface area, tunable pore structure, and adjustable chemical composition. These properties enable the modulation of MOFs’ electronic structures and facilitate charge transfer, demonstrating immense potential as catalyst supports in fuel cells. Through pyrolytic carbonization, MOFs can yield carbon materials with excellent conductivity or generate uniformly dispersed metal/metal oxide active sites, thereby enhancing catalyst utilization. In addition, MOFs can protect catalyst nanoparticles from agglomeration and leaching, contributing to the long-term stability of the catalyst. This protection can extend the catalyst’s lifetime and reduce the need for frequent catalyst replacement [59]. These advantages have led to the widespread application of MOF-derived materials in key reactions of fuel cells, such as the ORR and HOR. For instance, the ZIF-derived carbon material functions as a carrier due to its substantial specific surface area and distinctive structure and the considerable density of heteroatom doping and its defects. This facilitates the dispersion of platinum nanoparticles and enhances the interaction between the platinum nanoparticles and the carrier. Consequently, it improved the catalyst’s ORR intrinsic activity and stability [91]. Furthermore, the carbon layer derived from ZIF effectively suppresses the agglomeration and sintering of metal nanoparticles during the pyrolytic carbonization process, thereby preserving the small size and uniform dispersion of metal nanoparticles. This not only exposed more catalytically active sites but also improved the utilization rate of platinum, thereby further enhancing the catalytic activity and stability of the catalyst [92].

**Figure 11:**
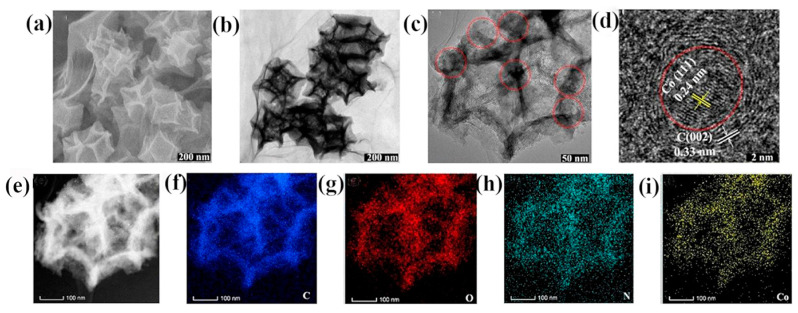
(**a**) SEM and (**b**) TEM images of NC@CoNC/rGO; (**c**,**d**) HRTEM image of NC@CoNC/rGO composites (red circles indicate the Co nanoparticles that are embedded in the carbon matrix); (**e**) EDS mapping image of an NC@CoNC/rGO composite; (**f**−**i**) EDS mapping images of elemental C (panel (**f**)), N (panel (**h**)), O (panel (**g**)), and Co (panel (**i**)) in the NC@CoNC/rGO composite [96]. Copyright 2020, American Chemical Society.

**Figure 12:**
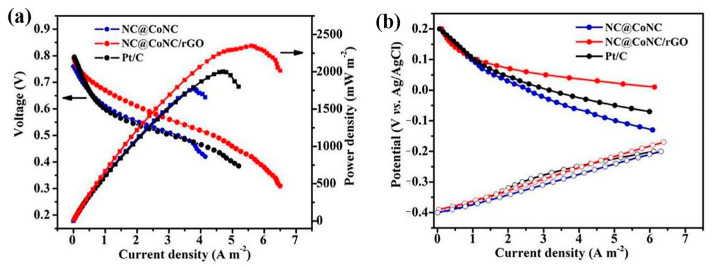
(**a**) Power density of MFC, (**b**) cathode and anode potential polarization curves with different cathode catalysts [96]. Copyright 2020, American Chemical Society.

Despite these advantages, MOF-derived carbon materials exhibit some limitations. For instance, nitrogen doping might adversely affect the electronic conductivity of carbon materials. It is crucial to strictly control the doping level to balance the electronic transport properties; otherwise, the overall performance of the catalyst may be compromised [93]. Zhou et al. [79] reported that the high-temperature synthesis of MOFs derived from carbon can lead to a large amount of nitrogen deficiency, which reduces the density of active sites and porosity, thus affecting catalytic activity. In this report, the ORR activity of FeCN with low active site density and poor porosity was poor, the E_onset_ was low at 0.92 V, and the E_1/2_ was low at 0.79 V. Fe-N-C with a high density of active sites and good porosity exhibited excellent ORR activity, with a high E_1/2_ of 0.83 V, which was only 20 mV lower than the benchmark Pt/C catalyst (E_1/2_, 0.85 V), and it was better than or equal to many of the most advanced Fe-N-C electrocatalysts (0.7 V < E_1/2_ < 0.86 V). In order to refrain from affecting the density of active sites and porosity, secondary nitrogen sources such as 2-dimethylimidazole (mIm) and 2,4,6-tris(2-pyridyl)-s-triazine could be introduced, and the pyrolysis temperature could be controlled [79,94,95]. In a previous report, Zhao et al. [96] pointed out that MOF-derived carbon materials have a low degree of graphitization, and the derived polyhedral particles tend to agglomerate together, resulting in poor catalyst performance. To overcome these drawbacks, the researchers composited MOF-derived core–shell carbon materials with conductive reduced graphene oxide (rGO) to form NC@CoNC/rGO composites. Figure 11 shows the electron microscopic characterization of the NC@CoNC/rGO composite. As shown in Figure 11a, the NC@CoNC/rGO composite has a typical layered structure. The snowflake-like rGO sheets can be seen in the TEM image in Figure 11b–d, showing that Co nanoparticles are encapsulated in the graphitic carbon layer, and the distance between neighboring grains was 0.24 nm, which agrees very well with the (111) facet of Co. In contrast, the specular spacing of 0.33 nm in the outer shell agrees with the (002) facet of graphitic carbon. Figure 11e–i revealed that C, N, O, and Co elements have good distributions in the material, and these structural features were favorable for improving its electrocatalytic performance. The experimental results in MFC showed that this composite possesses a maximum power density of 2350 mWm^−2^, which was 17% higher than that of the superior commercial Pt/C catalyst (2002 mWm^−2^), as shown in Figure 12a. The polarization curve of individual electrodes reveals that the anode potentials of all three MFCs behave similarly (Figure 12b). In contrast, the cathode potentials vary distinctly with increased current densities, suggesting that the cathode performance played an important role in MFC outputs. Therefore, the above results demonstrated that NC@CoNC/rGO catalysts have better electrocatalytic activity compared to the Pt/C catalyst, which showed promise as a good alternative catalyst with high power production and long-term stability in MFCs.

In summary, the unique properties of MOFs and their derivatives render them highly valuable and promising for research and applications in fuel cells whether as catalysts or catalyst supports. Overcoming challenges such as their limited conductivity, as well as the aggregation and uncontrolled dispersion of active sites and metal or metal oxide species, is crucial for realizing the full potential of MOFs and their derivative materials in fuel cell applications.

## 5. MOF- and Derivative-Modified Fuel Cell Membranes

MOFs and their derivatives are widely used to modify fuel cell membranes, in addition to being used as fuel cell catalysts. Ponnada et al. [97] presented recent advances in MOF-based membranes to enhance DMFC performance. Among the main findings was that composite membranes modified with MOFs have enhanced proton conductivity and reduced methanol permeability. They mainly use the following strategies to modify the fuel cell membrane: direct blending, in situ growth, and post-treatment methods. Each approach has advantages and disadvantages, making it suitable for different applications. The direct mixing method is the simplest and most commonly used for modifying fuel cell membranes with MOFs and their derivatives. For example, Gorban et al. [98] prepared sulfonated multi-block copolymer membranes (SPES) modified with HKUST-1 using the direct mixing method. They found that the addition of 5 wt% HKUST-1 significantly increased the membrane’s water absorption and ion exchange capacity while maintaining good mechanical properties and thermal stability. In situ growth can avoid the aggregation of MOFs and their derivatives during the mixing process, thereby enhancing the performance of fuel cell membranes. For instance, Chen et al. [99] incorporated SA-Hf-Uio-66-(OH)_2_ and CBD-Hf-Uio-NH_2_ into a chitosan matrix via in situ growth, preparing a fuel cell membrane with excellent proton conductivity. Their research showed that the introduction of MOFs significantly improved the proton conductivity of the membrane, with a maximum value exceeding 10^−2^ S cm^−1^. The post-treatment method allows for the flexible adjustment of the amount and distribution of MOFs and their derivatives, but a potential drawback is that it may affect the overall performance of the membrane. Soleimani and colleagues [100] used this method to incorporate ZIF-90 into sulfonated poly (1,4-phenylene ether-ether-sulfone) (SPEES) membranes, producing nanocomposite membranes with high proton conductivity and good mechanical properties. In addition, Ray’s team [101] performed the in situ polymerization of 1-vinyl imidazole using in situ polymerization and cross-linking techniques in the pores of MOF-808 and used N, N′-methylene bisacrylamide as a cross-linking agent to form cross-linked poly(1-vinyl imidazole) (CL-PVIMI); moreover, they added CL-PVIMI/MOF-808 composite fillers in different weight percentages. The CL-PVIMI/MOF-808 composite filler was added to the sulfonated polyether ether ketone (SPEEK) matrix at 2% and 4% to prepare a hybrid matrix membrane. The resultant membrane demonstrated a proton conductivity of 0.05 S·cm^−1^ at 80 °C and 70% relative humidity, which was 16.6 times higher than that of the pure SPEEK membrane. Notably, even at 100 °C and 40% relative humidity, proton conductivity remained at 0.04 S·cm^−1^. The incorporation of MOF into the matrix membrane led to substantial enhancements in both thermal stability and mechanical properties. Furthermore, electrochemical property tests demonstrated that the peak power density of fuel cells operating with S4P3M membranes (incorporating 4% composite filler) reached 310 mW/cm^2^ at 60 °C and 347 mW·cm^−2^ at 80 °C, respectively, thereby exhibiting superior performance in comparison to numerous SPEEK-based membranes that have been previously reported.

**Figure 13:**
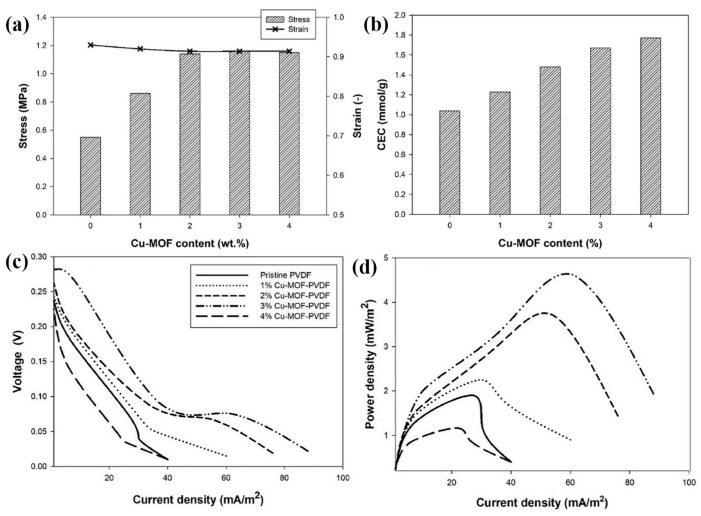
(**a**) Mechanical test result of the pristine (0 wt%) and Cu-MOF-doped (1–4 wt%) membranes. (**b**) The cation exchange capacity of the membranes. The voltage (**c**) and power density (**d**) versus current density results depending on the Cu-MOF concentration in the membrane [106]. Copyright 2024, Elsevier.

The effect of MOFs and their diversity as a pendant moiety on the polymer backbone had a significant effect on properties such as water uptake; thermal, mechanical, and oxidative stabilities; swelling ratio; ion-exchange capacity (IEC); morphology; proton conductivity; and fuel-cell performance [102]. Ru et al. [103] reported a mixed membrane, MNS@SNF-PAEK, in which a bifunctional metal–organic framework (MNS) was used as an inorganic nanofiller. This mixed membrane exhibited excellent mechanical strength, methanol resistance, and higher proton conductivity than pure SNF-PAEK membranes and commercial Nafion. Mukhopadhyay et al. [104] developed proton exchange membranes (PEMs) by incorporating synthetically rested UiO-66-NH_2_ metal–organic frameworks into an aryl ether-based polybenzimidazole (OPBI) polymer matrix. Subsequently, the composite membrane was doped with phosphoric acid (PA) to enhance its proton conductivity. The incorporation of thermally stabilized and hydrophilic MOF resulted in higher proton conductivity, higher PA retention capacity, and higher antioxidant stability compared to the pure OPBI polymer. The composite films exhibited significantly higher proton conductivity (0.29 S·cm^−1^ for PSM 1–10% and 0.308 S·cm^−1^ for PSM 2–10% at 160°C under anhydrous conditions), which is superior to most of the other MOF-based polymer composite films reported in the literature. Mahimai et al. [105] used Cu-MOF as a “nanocage” to encapsulate 1-methyl-3-propylimidazolium ionic liquid (IL), creating an IL-impregnated MOF (ML), which was then doped into sulfonated polystyrene-block-poly(ethylene-ran-butylene)-block-polystyrene (s-PSEBS) to form the S-PAEBS/ML composite membrane. Characterization and testing results showed that the addition of ML to the S-PSEBS polymer effectively enhanced the membrane’s proton conductivity and oxidative stability. Two years later, Nigiz et al. [106] reported the use of copper-based metal–organic frameworks (Cu-MOF) doped into polyvinylidene fluoride (PVDF) membranes to improve hydrogen transport and power generation performance in microbial fuel cells. From Figure 13a,b, it can be seen that the results of the study show that the doping of Cu-MOF increases the IEC from 1.04 mmol·g^−1^ to 1.77 mmol·g^−1^ and the mechanical strength from the original 0.55 MPa to 1.15 MPa. Since there is no equal charge distribution throughout the membrane, proper proton transfer and electricity production do not occur. In this study, the output voltage values obtained with 4 wt% of Cu-MOF-loaded membranes were lower than those of the pristine membrane (Figure 13c). This showed that excessive Cu-MOF incorporation in the membrane negatively affects electricity production. Figure 13b shows the power density versus current density. The highest power density of 4.62 Mw·m^−2^ was obtained with a 3 wt% Cu-MOF-doped membrane.

## 6. Summary and Outlook

This review summarized the various applications of MOFs in fuel cells, including their roles as catalysts, catalyst supports, and membrane modification materials. The high porosity and crystallinity of MOFs enable outstanding performance in electrochemical reactions, particularly in ORR and HOR. By precisely controlling the pore structure and chemical composition of MOFs, their electronic structure can be optimized to promote charge transfer, thereby enhancing the efficiency and stability of the catalysts. Furthermore, MOF-derived carbon materials and active metal/metal oxide sites not only improve the stability of the catalysts but also prevent the aggregation and leaching of catalyst nanoparticles.

Despite the significant progress in applying MOFs in fuel cells, several challenges remain. Firstly, the insufficient conductivity and difficulties in electron transfer limit their direct application in electrocatalysis. Secondly, the structural stability of MOFs during electrochemical reactions, such as catalyst corrosion or structural changes, may lead to a decline in catalytic activity. To overcome these challenges, future research needs to focus on the following areas: Enhancing the conductivity and electron transport properties of materials is paramount. MOFs are a class of materials that exhibit tunable electron transport properties through incorporating conducting polymers, carbon materials, or metal nanoparticles. In addition to conductivity, the structural stability and durability of MOFs are critical factors in determining their practical applications. The conversion of MOFs into derivatives with enhanced stability, such as metal–carbon composites or metal oxides, can be achieved through pyrolysis, etching, or chemical modification. In the development of MOFs, a multidisciplinary approach integrating materials science, chemical engineering, and electrochemistry is essential for the prediction and design of high-performance MOFs. This approach utilized computational chemistry to promote innovative applications in fuel cells and other fields, facilitating multidisciplinary cross- and synergistic innovation.

In conclusion, the potential applications of MOFs and their derivatives in fuel cells are promising, but interdisciplinary research efforts are required to address existing scientific and technological challenges. With continued research and innovation, MOFs and their derivatives are expected to play a key role in future energy conversion and storage technologies.

## Figures and Tables

**Table 1:** Physio-electrochemical properties of catalysts for ORR [74]. Copyright 2022, Elsevier.

Catalysts	Electrochemical					Physical Property
	ORR Peak Potential/V	ORR Onset Potential/V	Non-Faradic Current/mA	ORR Peak Current/mA	R_ct_/Ω	Diffusion/×10^−3^ cm^2^·s^−1^
Control	−0.89	−0.7	2.3	0.2	250	7
Zr-MOF	−0.39	−0.081	3.7	0.8	21.5	23
Zr-Ni-MOF	−0.377	0.0027	4.8	1.3	11.2	25
10% Pt-C	−0.55	−0.28	4.2	0.5	15.6	9

## Data Availability

The data presented in this study are available upon request from the corresponding author.
